# Contrary effects of increasing temperatures on the spread of antimicrobial resistance in river biofilms

**DOI:** 10.1128/msphere.00573-23

**Published:** 2024-02-07

**Authors:** Kenyum Bagra, David Kneis, Daniel Padfield, Edina Szekeres, Adela Teban-Man, Cristian Coman, Gargi Singh, Thomas U. Berendonk, Uli Klümper

**Affiliations:** 1 Institute for Hydrobiology, Technische Universität Dresden, Dresden, Germany; 2 Indian Institute of Technology, Roorkee, Uttarakhand, India; 3 Environment and Sustainability Institute, University of Exeter, Exeter, United Kingdom; 4 Institute of Biological Research Cluj, NIRDBS, Cluj-Napoca, Romania; University of Wisconsin-Madison, Madison, Wisconsin, USA

**Keywords:** antimicrobial resistance, invasion, river biofilm, climate change, temperature, one health

## Abstract

River microbial communities regularly act as the first barrier of defense against the spread of antimicrobial resistance genes (ARGs) that enter environmental microbiomes through wastewater. However, how the invasion dynamics of wastewater-borne ARGs into river biofilm communities will shift due to climate change with increasing average and peak temperatures remains unknown. Here, we aimed to elucidate the effects of increasing temperatures on the naturally occurring river biofilm resistome, as well as the invasion success of foreign ARGs entering through wastewater. Natural biofilms were grown in a low-anthropogenic impact river and transferred to artificial laboratory recirculation flume systems operated at three different temperatures (20°C, 25°C, and 30°C). After 1 week of temperature acclimatization, significant increases in the abundance of the naturally occurring ARGs in biofilms were detected at higher temperatures. After this acclimatization period, biofilms were exposed to a single pulse of wastewater, and the invasion dynamics of wastewater-borne ARGs were analyzed over 2 weeks. After 1 day, wastewater-borne ARGs were able to invade the biofilms successfully with no observable effect of temperature on their relative abundance. However, thereafter, ARGs were lost at a far increased rate at 30°C, with ARG levels dropping to the initial natural levels after 14 days. Contrary to the lower temperatures, ARGs were either lost at slower rates or even able to establish themselves in biofilms with stable relative abundances above natural levels. Hence, higher temperatures come with contrary effects on river biofilm resistomes: naturally occurring ARGs increase in abundance, while foreign, invading ARGs are lost at elevated speeds.

**IMPORTANCE:**

Infections with bacteria that gained resistance to antibiotics are taking millions of lives annually, with the death toll predicted to increase. River microbial communities act as a first defense barrier against the spread of antimicrobial resistance genes (ARGs) that enter the environment through wastewater after enrichment in human and animal microbiomes. The global increase in temperature due to climate change might disrupt this barrier effect by altering microbial community structure and functions. We consequently explored how increasing temperatures alter ARG spread in river microbial communities. At higher temperatures, naturally occurring ARGs increased in relative abundance. However, this coincided with a decreased success rate of invading foreign ARGs from wastewater to establish themselves in the communities. Therefore, to predict the effects of climate change on ARG spread in river microbiomes, it is imperative to consider if the river ecosystem and its resistome are dominated by naturally occurring or invading foreign ARGs.

## INTRODUCTION

The rise of antimicrobial resistance (AMR) has been considered a silent pandemic on a global scale (1). Based on global clinical reports, 4.95 million people succumbed to bacterial infections with resistant pathogens in 2019 (2), with numbers expected to rise up to 10 million deaths per year by 2050 (3). Mitigating the spread of AMR is difficult as there is no clear barrier between the environment, animal, and human microbiomes (4). It is hence imperative to identify the barriers that could limit the spread of AMR within and between the microbiomes of these compartments (5–7).

Rivers and river biofilms could play a major role in the spread of AMR as rivers are not only one of the main sources of daily water supply but, in many parts of the world, also serve as a conduit of waste and wastewater from households, hospitals, animal, and aquatic farms (8–12). High loads of antibiotic-resistant bacteria (ARBs) and antibiotic-resistant genes (ARGs) are constantly transferred via fecal matter through wastewater streams to wastewater treatment plants (WWTPs) (13). In wastewater, ARBs and ARGs are also exposed to chemical pollutants like pharmaceuticals, pesticides, and heavy metals, which induce selection pressure and promote horizontal gene transfer, thus making WWTPs a hotspot for the spread of AMR (14–16). Despite a general reduction in total bacterial numbers through WWTPs, the discharge of treated wastewater into rivers still introduces significant loads of ARBs and ARGs to the receiving waters (17–19). Additionally, in many locations, wastewater is discharged without any prior treatment (20–22). This accounts for around 48% of the globally produced wastewater entering environmental waters untreated (23). Even in countries like Japan, with one of the highest WWTP coverage in the world at 91.4%, untreated water from around 14 million people is discharged into the environment (24). Moreover, extreme weather events like floods, heavy rainfall, monsoons, and cyclones, which are expected to occur at higher frequency due to climate change, result in the overflow of wastewater streams, which are then released into the environment untreated alongside the stormwater (11, 25–27).

The ARB discharged into the river must overcome the stress in the new environment as well as compete for available resources against the bacterial community already established in the river (28). This suggests that river microbial communities regularly act as the first barrier against the spread of ARB and ARGs that are enriched in the anthroposphere and enter the environment through wastewater (29). Unsurprisingly, such discharges of treated and untreated wastewater are often directly linked to increased levels of AMR in the microbiomes of receiving water bodies making it a crucial pathway for the dissemination of ARGs (9, 17, 30, 31). Consequently, understanding what drives the invasion success of ARB and ARGs from wastewater into river microbiomes is essential to combat their establishment in river microbiomes.

When wastewater enters rivers, the stress caused by the release of chemical pollutants into the environment can directly impact the structure of microbial communities (16, 32). Disturbances of the microbial community structure can pave the way for the successful establishment of incoming resistant bacteria into river biofilm communities (33). Exposure to stress is hence a defining factor in shaping the natural invasion barriers of environmental communities. A major stress factor that environmental microbiomes worldwide are currently adapting to is the increase in average and peak temperatures through climate change. According to the Intergovernmental Panel on Climate Change, the global temperature increased by 1°C since the pre-industrial era and a global temperature rise by more than 1.5°C from 2030 to 2052 (34) is to be expected. The rise in air temperature is likely to be accompanied by an increase in surface water temperatures as both are directly linked (35, 36). Many studies have reported resulting shifts in microbial community composition and function in soils, river waters and sediments (37–40).

In the context of AMR in river microbiomes, such a rise in temperature could result in two main outcomes: first, changes in community composition due to changing temperatures could directly lead to the enrichment or loss of ARG host bacteria, depending on their adaptation to the warming conditions. Second, warming could also affect how river microbial communities function as a barrier to invading ARBs from wastewater. During such invasion events, the interplay between different temperature optima of the resident community and the resistant invaders could affect their competitive interactions and hence the outcome of the invasion events (41). Furthermore, higher temperatures are regularly connected to higher metabolic and growth rates of all bacteria involved (42), which could intensify competitive interactions during the invasion process. Based on these predictions, we here determined if higher temperatures due to climate change could indeed affect AMR in river microbial communities by investigating temperature effects on both the intrinsic river biofilm resistome and the invasion success of foreign ARB and ARG entering through wastewater into these biofilms.

To achieve this, river biofilms were grown on glass slides immersed into a low anthropogenic impact sub-tributary of river Elbe in Germany for 1 month. Biofilms were then transferred to artificial laboratory recirculation river water flume systems. These flume systems were operated at three different temperatures of 20°C, 25°C, and 30°C. Biofilms were allowed to acclimatize for 1 week to the respective temperature. Thereafter, the biofilms were exposed to a single pulse of wastewater, and invasion dynamics were monitored for 2 weeks. Throughout the experiment, biofilms were destructively sampled, meaning that glass slides were completely removed from the flume system and the colonizing biofilms were harvested from these slides. The biofilm resistome was characterized using high throughput qPCR, while the biofilm microbial community composition was analyzed using 16S rRNA gene-based amplicon sequencing. This allowed determining the time-resolved invasion dynamics of wastewater-borne ARB and ARGs into the river biofilms as a function of temperature. In addition, control group flumes without wastewater addition were run to determine the effect of increasing temperatures on the natural microbiome and resistome composition.

## MATERIALS AND METHODS

### Biofilms and river water

Natural river biofilms were obtained from river Hirschbach (50° 54′17.6″N, 13° 45′ 06.3″E), which is a part of the Lockwitzbach sub-Elbe tributary in Saxony, Germany. The chosen location had no WWTP or agricultural fields located upstream and was hence of low anthropogenic impact. Biofilms were grown on rectangular glass slides of 76 × 26 mm size (DWK Life Science, Wertheim, Germany) that were mounted on artificial exposure units (AEUs) constructed from Plexiglas (Fig. S1). Twelve AEUs each containing nine glass slides were screwed onto a concrete plate and covered with a stainless-steel cover for protection and to provide shade to limit the growth of phototrophic organisms. A metal mesh at the front and back allowed river water to stream through the AEUs. AEUs were immersed in the river for 48 days in March 2022 to allow biofilm growth. The AEUs containing the glass slides with grown biofilms were then collected and individually transferred to the laboratory in a 1 L box containing river water at ambient temperature. Simultaneously, 100 L of the same river water was collected, immediately transported to the laboratory, and filter sterilized using 0.2-µm pore size membrane filters (Whatman, Maidstone, UK) for use in the artificial flume systems.

### Laboratory flume system

Laboratory artificial flume systems (33) with a dimension of 40 × 19.1 × 10 cm (L × W × H) were constructed from polypropylene with an inlet and an outlet attached to each end (Fig. S1). The inlet and outlet of the flume were connected to a 2 L reservoir, which was attached to a recirculation pump. Flumes were filled with a total volume of 6.348 L sterile river water constantly recirculated with the pump at 100 mL/min. The inlet was 1 cm in diameter and at 0.5 cm height, a PVC pipe was inserted on the inner side of the system pointing toward the bottom of the flume to allow for an even distribution of the water flow inside the flume system. The outlet was at the back, located at 7 cm height to maintain a constant maximum water level inside the flume. The artificial flumes were kept covered with polypropylene lids to avoid light exposure. Three AEUs containing glass slides with grown biofilms were placed in the middle of each flume filled with sterile filtered river water from the site.

### Wastewater sampling

Wastewater was collected from the influent of the WWTP located at Dresden-Kaditz, Germany (51.07 °N, 13.67 °E) on 21 March 2022, in a sterile 10 L bottle. Suspended solids and sand particles were allowed to settle for 2 min, and the resulting supernatant was used for flume experiments after any large debris still floating in the wastewater that could clog the flume system was removed manually with sterilized tweezers. For subsequent analysis of the bacterial communities of the wastewater, 50 mL of the supernatant wastewater was filtered in triplicate with 0.2-µm pore size membrane filter paper (Whatman, Maidstone, UK), and the filters were stored at −20°C for downstream DNA extraction and analysis.

### Flume experiments

Two artificial flume systems containing AEUs with grown river biofilms were placed in each climate chamber set to 20°C, 25°C, and 30°C, respectively. Temperature was monitored throughout the experiment and remained constant (± 1°C). Before starting any treatment, biofilms were allowed to acclimatize in the recirculation flumes to the laboratory conditions for a week. After this period, one of the flumes from each chamber was inoculated with wastewater. A single pulse of 1.375 L (20% of flume volume) of influent was added while simultaneously removing excess water from the outlet. The other flume at each temperature was used as the control, where instead of wastewater, 1.375 L of sterile river water was added. The flumes were then run for 14 days at stable conditions.

### Biofilm sampling

Biofilms were destructively sampled right before adding the wastewater to assess the initial diversity after acclimatization of the microbiome (T0). Furthermore, biofilms were destructively sampled on days 1, 4, 7, and 14 after the influent was added. For each time point, an individual glass slide with biofilm was randomly removed from each of the three AEUs of both the influent and control flume from each of the chambers, resulting in three biological replicates per time point and treatment. The biofilm slides were picked randomly after generating the order of removal for each individual AEU using a random number generator in R studio (43). Each individual biofilm slide was carefully placed inside a 50 mL centrifuge tube with 1 mL of sterile detergent solution (0.9% NaCl solution with 0.05% Tween80 [Sigma-Aldrich, St. Louis, MO, USA]). Biofilms were scraped from the glass slide into the tube using a cell scraper (TPP, Trasadingen, Switzerland). The scraper and the glass slide were washed with the detergent solution to allow complete detachment of the biofilm. After discarding the empty glass slide, the collected biofilms were centrifuged at 4,000 rpm for 10 min, and the supernatant liquid was carefully removed through pipetting. The resulting biofilm pellet was weighed and stored at −20°C until subsequent DNA extraction.

### DNA extraction

DNA extraction from biofilm and wastewater samples was carried out using the Qiagen DNeasy PowerSoil Pro Kit (Qiagen, Hilden, Germany) as per the manufacturer’s instruction. Sufficient quality and quantity of extracted DNA were assessed using a NanoDrop One spectrophotometer (Thermo Fisher Scientific, Waltham, MA, USA).

### High-throughput qPCR analysis for ARGs, MGEs, and taxonomic markers

To determine the relative abundance of target genes in the biofilm and wastewater samples, DNA extracts were sent to Resistomap Oy (Helsinki, Finland) for HT-qPCR analysis using a SmartChip Real-Time PCR system (TaKaRa Bio, Shiga, Japan). A total of 10 ng DNA of each of the extracted samples were shipped to Resistomap for analysis. The target genes were quantified based on previously published primers (44) and included 27 ARGs and 3 mobile genetic element (MGE) markers, along with the house-keeping gene, the 16S rRNA gene, for the normalization of relative abundances ( Table S1). Two fecal indicator taxonomic markers (*Escherichia coli* and *Enterococcus*) were quantified additionally. The PCR mixture (100 nL) was prepared using SmartChip TB Green Gene Expression Master Mix (TaKaRa Bio), nuclease-free PCR-grade water, 300 nM of each primer, and 2 ng/µL DNA template alongside a nuclease-free PCR-grade water-based negative control. After initial denaturation at 95°C for 10 min, PCR comprised 40 cycles of 95°C for 30 s and 60°C for 30 s, followed by melting curve analysis for each primer set. Amplicons with non-specific melting curves or multiple peaks were excluded. The relative abundances of the detected gene to the 16S rRNA gene were estimated using the ΔCT method based on mean CTs of three technical replicates (45). Any results with multiple peaks and amplification efficiency outside the range of 0.9–1.1 were discarded. The threshold cycle (Ct) of 31 was set as the limit of quantification (LOQ). The gene copies were calculated using equation 1 (46)


(1) $$Gene\;copy\;number=10^{\left((31-Ct)/(log(10)/log(2))\right)}$$

where Ct is the threshold cycle of the qPCR result, 31 is the LOQ-Ct, and log (10)/log (2) refers to the number of cycles of the 10-fold difference in the gene copy numbers, when the efficiency is 100%.

### Absolute bacterial abundance

To determine the absolute bacterial abundance on the biofilm carrier slides, the bacterial 16S rRNA marker gene was quantified using real-time qPCR in a C1000 Touch Thermal cycler (Biorad, Hercules, CA, USA) and the 338F (CCTACGGGAGGCAGCAG) 518R (ATTACCGCGGCTGCTGG) primer set (47). As a qPCR standard, the recombinant plasmid pBELX-1 (48) was extracted using the Wizard plus SV Miniprep DNA Purification System (Promega, Madison, WI, USA) according to the manufacturer’s instructions and linearized by restriction enzyme BamHI (Promega) before purification with the QIAquick PCR purification kit (Qiagen). Reactions were performed in technical triplicates in a MasterCycler RealPlex (Eppendorf, Hamburg, Germany) at a final volume of 20 µL with 10 µL of Luna Universal qPCR Master Mix (New England Biolabs, Frankfurt, Germany). Each primer was added at a final concentration of 300 nM, and the reactions were run with 10 ng of DNA extract. The PCR program consisted of initial denaturation at 95°C for 10 min and 40 cycles of denaturation (95°C; 15 s) and annealing and elongation (60°C; 1 min). Standard curves for the targets were created during every qPCR run, using the above-described standard plasmid with the standard target concentrations ranging from 10^6^ to 10^1^ copies per reaction. Standard curves with amplification efficiency 0.9–1.1 and *R*^2^ ≥ 0.99 were accepted, and melting curve analysis was performed to assess the amplicons’ specificity. Screening for PCR inhibition was performed by spiking the standard plasmid into the DNA samples. No inhibition was detected in any of the samples. The limit of quantification was calculated for each individual qPCR run according to the MIQE guidelines (49).

### 16S sequencing and analysis of microbial community diversity

The microbial community diversity and structure were profiled using high-throughput amplicon sequencing of the V3–V4 region of the 16S rRNA gene. DNA extracts were sent to IKMB Kiel University (Germany), and the partial 16S rRNA genes were sequenced on an Illumina Novaseq using the primers (v3f: CCTACGGGAGGCAGCAG; v4r: GGACTACHVGGGTWTCTAAT). Sequence analysis was carried out using Mothur v.1.37.1 (50) according to the MiSeq SOP (51) as accessed on 03 July 2022. Sequences were classified based on the RDP classifier (52). All sequencing data presented in this paper have been submitted to the NCBI sequencing read archive under the accession number PRJNA1014398.

### Identification of invasion indicators

To investigate the invasion success of wastewater-borne ARGs and bacteria, invasion indicators were defined. Potential invasion indicators were defined as those targets from the HT-qPCR or the operational taxonomic unit (OTU)-level analysis that have a significantly higher relative abundance in wastewater influent samples compared to the biofilm samples at T0 and would hence be expected to rise upon exposure to the wastewater in the biofilms. Successful invasion indicators were then defined as the fraction of potential indicators that did indeed significantly increase in the exposed biofilms at T1 when compared to the control treatment. To determine significance, multilevel pattern analysis was carried out based on the relative gene abundances from the HT-qPCR assay and the relative OTU abundance from the community diversity analysis using the multipatt function of the indicspecies package in R (53). For every mentioned computation, the grouped biserial correlation and the phi correlation coefficients were calculated using a complex permutation design (*n* = 9,999) using the permute package in R (54). Using this setup, finally 6 of the 27 ARGs, 1 of the 3 MGEs, and both fecal indicator taxonomic targets were defined as indicators of invasion success over time and used to determine the temperature effects on invasion. Furthermore, among the total of 67,502 identified bacterial OTUs from diversity analysis, 235 were defined as indicators of bacterial invasion success over time and equally used to determine temperature effects on invasion dynamics.

### Statistical analysis

All statistical analysis was performed in R Studio v4.1. (43). The relative abundance of each target gene derived from HT-qPCR was compared between control and exposed biofilms at timepoints T0, T1, T4, T7, and T14 for each temperature group using a Wilcoxon rank-sum test. *P*-values were adjusted using the Benjamini and Hochberg method. Relative abundances below the quantification limit were set as “<LOQ” and as the lowest rank in the rank-sum evaluation when comparing it to samples with relative abundances above the LOQ before computation.

A pairwise comparison of the sum of the abundance of all ARGs in the biofilms at T0 between different temperatures was tested using the Student’s *t*-test. The change over time in the sum of the relative abundance of all ARGs was tested for each treatment using the Kruskal-Wallis test. To test if the relative abundance of each individual ARG across different temperatures in the control flumes follows a global trend with temperature, the Wilcoxon rank-sum test was applied.

Trends regarding the relative abundance of target genes over time were tested based on Pearson correlation coefficients using the “ggplot” package (55). To test if the slopes of the linear regression for the individual indicator genes over time follow a global trend based on temperature, the Wilcoxon rank-sum test was applied.

Changes in bacterial diversity were assessed based on observed OTUs at 97% sequence similarity using the Bray-Curtis dissimilarity metric in the “vegan” package (56). Differences between the biofilm and wastewater community structure of control and exposed groups at different temperatures were tested using ADONIS in R v4.1.0 under the “vegan” package (56). Difference in the phylum level abundance between the biofilm community at T0 and the wastewater community was tested using the Kruskal-Wallis rank sum test.

Throughout, a *P*-value < 0.05 was defined as a statistically significant observed effect.

## RESULTS

### Distinct river microbial biofilm communities based on incubation temperature

To study the effect of rising temperatures on AMR dynamics in rivers, river biofilms were grown on glass slides and set to acclimatize for 1 week at three climate chambers of temperature 20°C, 25°C, and 30°C. Thereafter, the biofilms for each temperature were subjected to two individual treatments: one group was exposed to wastewater, and a control group remained unexposed, with the experiment lasting for 14 days.

**Fig 1:**
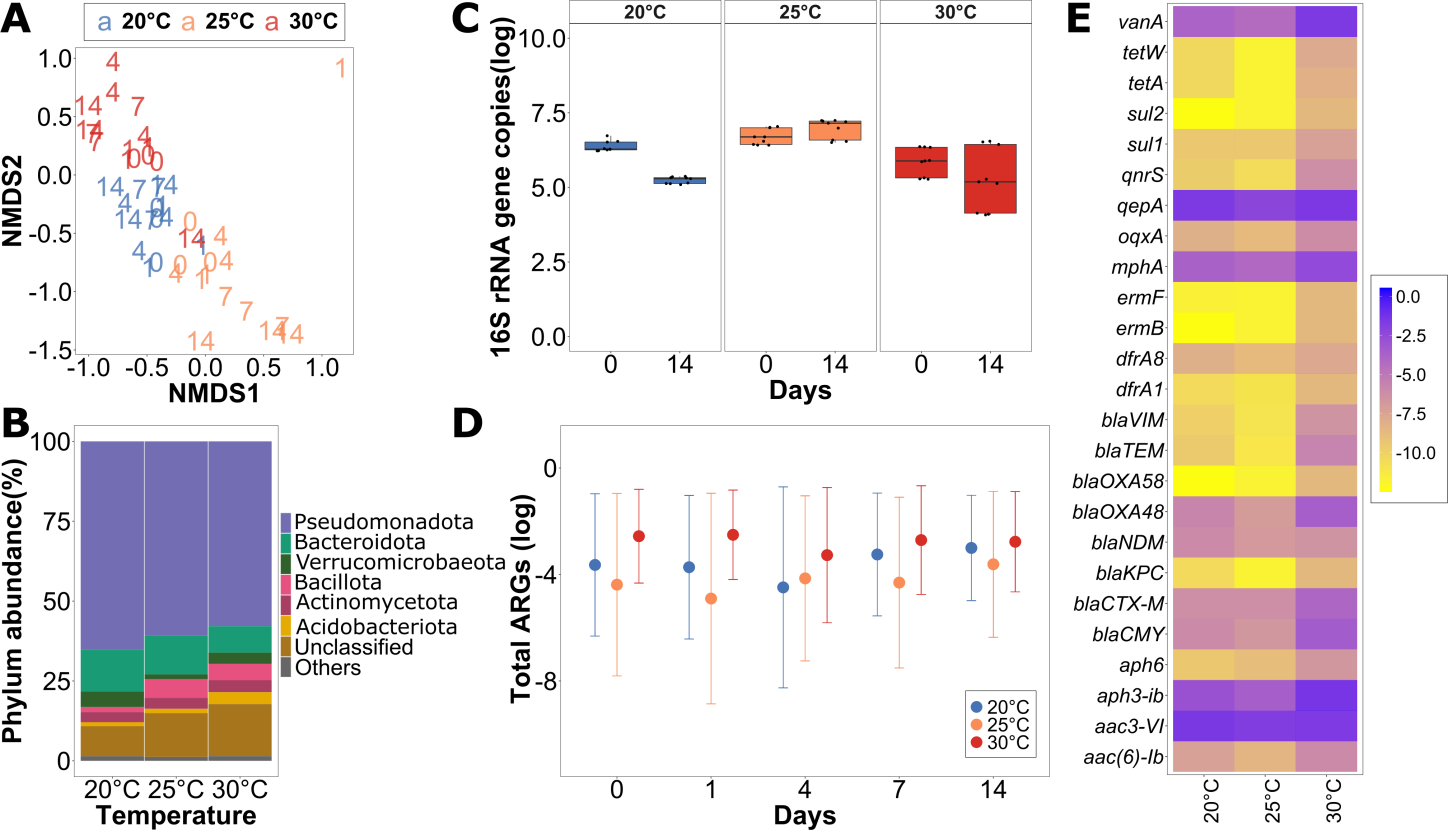
The initial diversity of the control biofilms after 1 week of acclimatization to the laboratory condition with different temperatures. (**A**) NMDS (non-metric multidimensional scaling) plot using the Bray-Curtis dissimilarity to analyze the beta-diversity of the biofilm for the control group for T0, T1, T4, T7, and T14 for all the temperature groups. (**B**) Relative phylum abundance in percentage of the biofilms at T0 across all the temperature groups. (**C**) Initial and final abundances of absolute 16S rRNA gene copies per cm^2^ in the biofilms across all temperature groups. (**D**) Average of the sum of all tested ARG relative abundances normalized per 16S rRNA gene copies present in the biofilms for T0, T1, T4, T7, and T14 in the control group throughout the experiment across all three temperatures. The error bars represent the standard deviation. (**E**) Heatmap showing the initial relative abundance of ARGs present in biofilms in the control group for different temperature groups.

To determine if temperature on its own influenced the microbial community and resistome composition, biofilms from the control group were investigated first. At the initial timepoint (T0) after completing the 1-week acclimatization period, river microbial communities clustered distinctly based on the incubation temperature (Fig. 1A). Replicate biofilms from flumes incubated at the same temperature clustered significantly together and apart from those incubated at different temperatures (*P* < 0.001, *R*^2^ = 0.37, ADONIS). Still, the most dominant phyla in the biofilms from all three temperatures were consistently *Pseudomonadota* (57.87% ± 12.39% to 65.29% ± 7.50%), *Bacteroidota* (8.27% ± 2.41% to 13.1% ± 2.19%), and *Bacillota* (5.86% ± 6.75% to 1.59% ± 1.03%) (Fig. 1B). While a minor change in community composition was observed over time, the grouping based on temperature stayed consistent for the duration of the experiment (*P* < 0.001, *R*^2^ = 0.29, ADONIS) (Fig. 1A). Furthermore, no statistically significant trend regarding the total amount of biomass based on 16S rRNA gene copies per cm² of the biofilm carrier was observed during the 14 days of recirculation in the flume. Bacterial numbers remained within the same order of magnitude throughout the experiment, suggesting that growth is a negligible factor in the experiment (Fig. 1C).

### Characterization of the invading wastewater community

**Fig 2:**
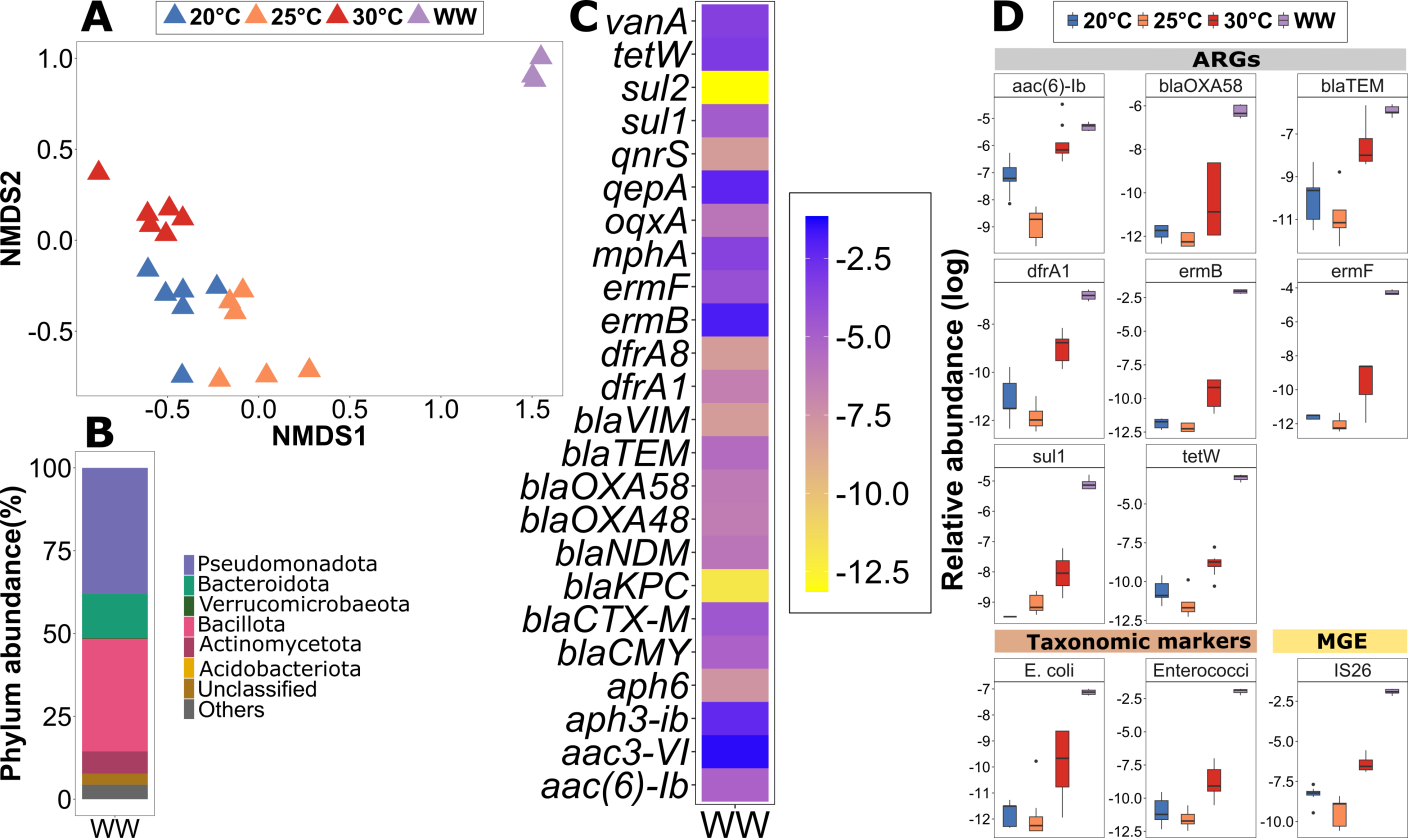
Wastewater and biofilm composition (T0) and potential indicators of invasion before inoculation. (**A**) NMDS (non-metric multidimensional scaling) plot using the Bray-Curtis dissimilarity at the OTU level to analyze the diversity of wastewater and biofilms at T0 (stress = 0.1667). (B) Relative phylum abundance in percentages of the wastewater. (**C**) Relative abundance of all the ARGs tested in wastewater before inoculation. (**D**) Boxplot showing the initial relative abundances of ARGs, an MGE, and two taxonomic markers identified as potential indicators of temperature groups at T0 and wastewater used for inoculation (*n* = 9).

The wastewater microbial community was significantly dissimilar from the biofilm communities at T0 (*P* < 0.001, *R*^2^ = 0.29, ADONIS) (Fig. 2A). While the three dominant phyla, *Pseudomonadota* (38.04% ± 27.8%), *Bacillota* (33.95% ± 0.61%), and *Bacteroidota* (13.26% ± 1.2%) were similar to the biofilms, significantly more *Bacillota* and significantly less *Pseudomonadota* were observed in the wastewater (Fig. 1B and 2B) (*P* < 0.05, Kruskal-Wallis rank sum test).

### Elevated ARGs at higher temperatures in river biofilms

To determine if not only community composition but also the resistome of the river biofilm communities were altered due to increasing temperatures, HT-qPCR of 27 ARGs was performed. The most abundant ARGs present in the control flume biofilms across temperatures were *aac3-VI* (0.15 ± 0.07 to 0.63 ± 0.19 ARG copies/16S copies), *qepA* (0.13 ± 0.05 to 0.35 ± 0.09), *aph3-ib* (0.08 ± 0.07 to 0.36 ± 0.34), *mphA* (0.024 ± 0.01 to 0.11 ± 0.02), and *vanA* (0.03 ± 0.02 to 0.17 ± 0.05) (Fig. 1E). These confer resistance to aminoglycosides, quinolones, macrolides, and vancomycin. While the same set of ARGs was dominant across temperatures, the sum of all relative abundances of all the targeted ARGs was significantly higher in the biofilms exposed to a temperature of 30°C with 1.82 ± 0.16 ARGs per 16S rRNA gene (Fig. 1E) compared to 20°C (0.60 ± 0.27, *P* < 0.01, paired *t*-test) and 25°C (0.28 ± 0.05, *P* < 0.01). However, there was no significant difference between the two lower temperatures (*P* = 0.354). This trend was mirrored at the individual ARG level, where an increase in relative abundance with temperature was observed for a significant proportion of individual ARGs at 30°C compared to 20°C (*P* = 0.003) and 25°C (*P* < 0.001) based on a Wilcoxon rank-sum test. Similar to the microbial community composition, the relative abundance of ARGs for the control group did not undergo any significant changes over the 14 days of flume experiment (*P* > 0.2, Kruskal-Wallis test). However, the total ARG abundance at 30°C remained significantly elevated at all time points compared to 20°C and 25°C (*P* < 0.05, Kruskal-Wallis test) (Fig. 1D). This indicates that higher temperatures select for those community members that are hosting more ARGs initially, but that communities and their resistome are not affected by temperature once they reach a stable state after acclimatization.

### Identification of invasion indicator ARGs from wastewater into the river biofilm community

To quantify the invasion success of wastewater-derived ARGs into the biofilm community, potential invasion indicators were defined as those targets from the HT-qPCR analysis that have a significantly higher relative abundance in wastewater compared to the biofilm samples at T0 and would hence be expected to rise in relative abundance in the biofilms upon exposure to the wastewater.

Among the 27 tested ARGs, those most abundantly identified in the biofilms at T0 were also most abundant in the wastewater: *aac3-VI* (0.25 ± 0.03 ARG copies/16S copies), *qepA* (0.1 ± 0.01), *aph3-ib* (0.08 ± 0.01), *mphA* (0.03 ± 0.003), and *vanA* (0.04 ± 0.004) (Fig. 2D). Other abundant ARGs in wastewater were *ermB* (0.13 ± 0.02), *tetW* (0.04 ± 0.006), and *ermF* (0.014 ± 0.001). These ARGs confer resistance to multiple antibiotic classes including aminoglycosides, quinolones, macrolides, vancomycin, and tetracyclines, respectively.

Still, significant difference in relative abundance between wastewater and biofilms was observed. A total of eight ARGs (*ermF*, *ermB*, *tetW*, *sul1*, *blaOXA58*, *dfrA1*, *blaTEM*, and *aac.6.Ib*) were identified as significantly more abundant in wastewater using multilevel pattern analysis (*P* < 0.01) (Fig. 2D) and selected as potential indicators of invasion. Additionally, two fecal bacterial indicators, *E. coli* and *Enterococci,* and one MGE (*IS26*) displayed a significantly higher association with the wastewater compared to the initial biofilms at all temperatures (*P* < 0.01) (Fig. 2D). An observable increase in relative abundance of these prevalent target genes in the biofilm upon exposure to wastewater would hence provide evidence of successful invasion events into the river biofilms.

### Successful and temperature-independent invasion of ARGs from the wastewater into the river biofilm community

**Fig 3:**
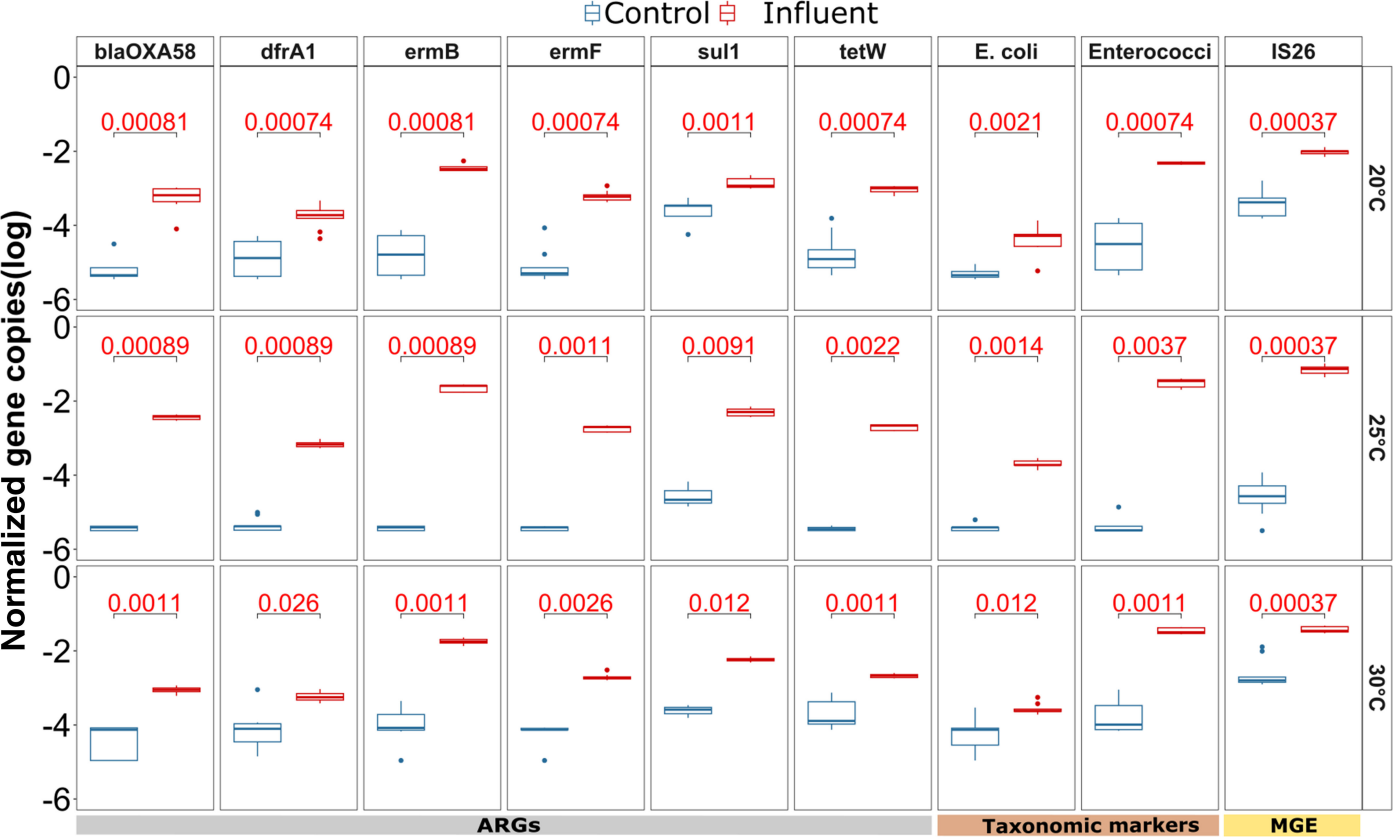
Identification of indicator ARGs, taxonomic markers, and MGEs for which a significant difference in relative abundance (log) was observed on day 1 after inoculation, compared between the wastewater exposed and the control biofilms across the three different temperatures (20°C, 25°C, and 30°C). The *P*-value (Wilcoxon signed rank test) of the comparison for each gene (*n* = 9), after applying Benjamin-Hochberg correction for multiple testing, is displayed at the top of each plot, with significant values (*P* < 0.05) displayed in red.

To determine if invasion was indeed taking place, the abundance of the identified indicators in the river biofilms exposed to wastewater from all three temperature groups was tested 1 day after inoculation of the wastewater. Successful invasion indicators were then defined as the fraction of potential indicators that did indeed significantly increase in the exposed biofilms at T1 when compared to the control treatment. After 1 day, six of the eight ARGs (*blaOXA58, dfrA1, ermB, ermF, sul1,* and *tetW*) were significantly more abundant in the biofilm that was exposed to wastewater compared to those in the control flumes for each of the three different temperatures (all *P* < 0.05, Wilcoxon test) (Fig. 3). Especially, *ermB, ermF,* and *blaOXA58*, which were below the detection limit in the majority of replicate biofilms from the control samples, had high levels at T1 in the exposed biofilms indicating successful invasion from wastewater. After day 1 of invasion, for each of these six indicator ARGS, the relative abundance level was within one order of magnitude among different temperatures. Moreover, there was no clear trend observed with regard to the initial invasion efficiency across the different temperatures based on a Wilcoxon test.

Notably, the remaining two ARGs significantly associated with wastewater (*blaTEM* and *aac.6.Ib*) did not significantly increase in abundance across all temperatures (Fig. S2), indicating that the invasion of these indicator ARGs is not merely stochastic but potentially connected to the specific ARG hosts that might have different invasion efficiency into the biofilm microbiome. Importantly, for the remaining 19 ARGs, where the relative abundance in the wastewater and the T0 biofilm communities displayed no significant difference and that were hence previously excluded as potential indicators of invasion, no consistent significant increase across all temperatures was observed (*P* > 0.05 for at least one temperature per ARG, Wilcoxon test; Fig. S2). Among the three potential non-ARG indicators, the MGE *IS26* (*P* < 0.001, Wilcoxon rank sum test), *E. coli* (*P* < 0.05, Wilcoxon rank sum test), and *Enterococci* (*P* < 0.001, Wilcoxon rank sum test) showed a significant increase on day 1 after inoculation for all three temperatures (Fig. 3). In summary, a clear indication of successful initial invasion by ARGs or ARBs from wastewater into the biofilms independent of temperature was detected right after the introduction on day 1, which allows investigating the fate of these invaders from wastewater over time as a function of temperature.

### Invasion indicator genes and organisms are lost more rapidly with increasing temperature

**Fig 4:**
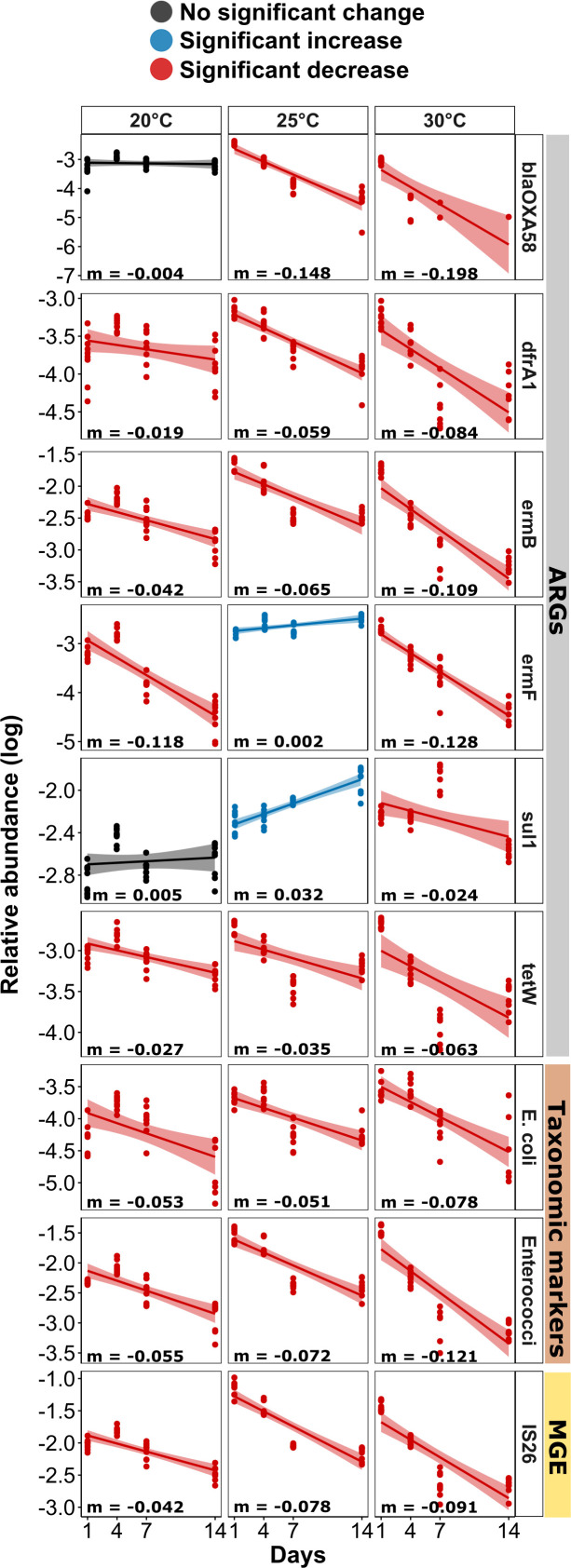
Temporal dynamics of the nine identified indicator genes in wastewater-invaded biofilms across three temperature groups. Linear regression for the 16S rRNA gene normalized relative indicator gene abundance (log transformed). Red lines indicate a significant decrease, black lines indicate no significant change, and blue lines indicate a significant increase of the marker gene over time based on Pearson correlation analysis (*P* < 0.05).

To understand the fate of the invaders over time after initially entering the biofilms, biofilms were sampled on days 1, 4, 7, and 14. At the highest temperature (30°C) for all nine indicators (six ARGs, one MGE, *E. coli*, and *Enterococci*), a clear decline in abundance with a significantly negative slope based on Pearson correlation was observed (all *P* < 0.05; Fig. 4). While similarly declines in abundance over time were observed for the majority of indicators at 20°C and 25°C, the slopes were less steep, demonstrating a lower loss rate of indicators over time (Fig. 4). This manifested statistically, as overall, the loss rate of each of the invading nine indicator genes and organisms over time was significantly higher at 30°C compared to 20°C and 25°C (both *P* < 0.01) based on a Wilcoxon signed-rank test for temperature, while no significant difference in loss dynamics was observed between the two lower temperatures. In certain cases, such as *blaOXA58* (*P* = 0.67, Pearson correlation) and *sul1* (*P* = 0.50) at 20°C, there was no significant decrease in abundance over time observed for the invading ARGs, and their abundance remained stagnant. The genes *sul1* and *ermF* even slightly but significantly increased over time at 25°C (both *P* < 0.05). Contrary to the wastewater-inoculated flumes, not a single significant decrease over time was detected in the control treatment for either of the indicator genes at any temperature (all *P* > 0.05; Fig. S3).

**Fig 5:**
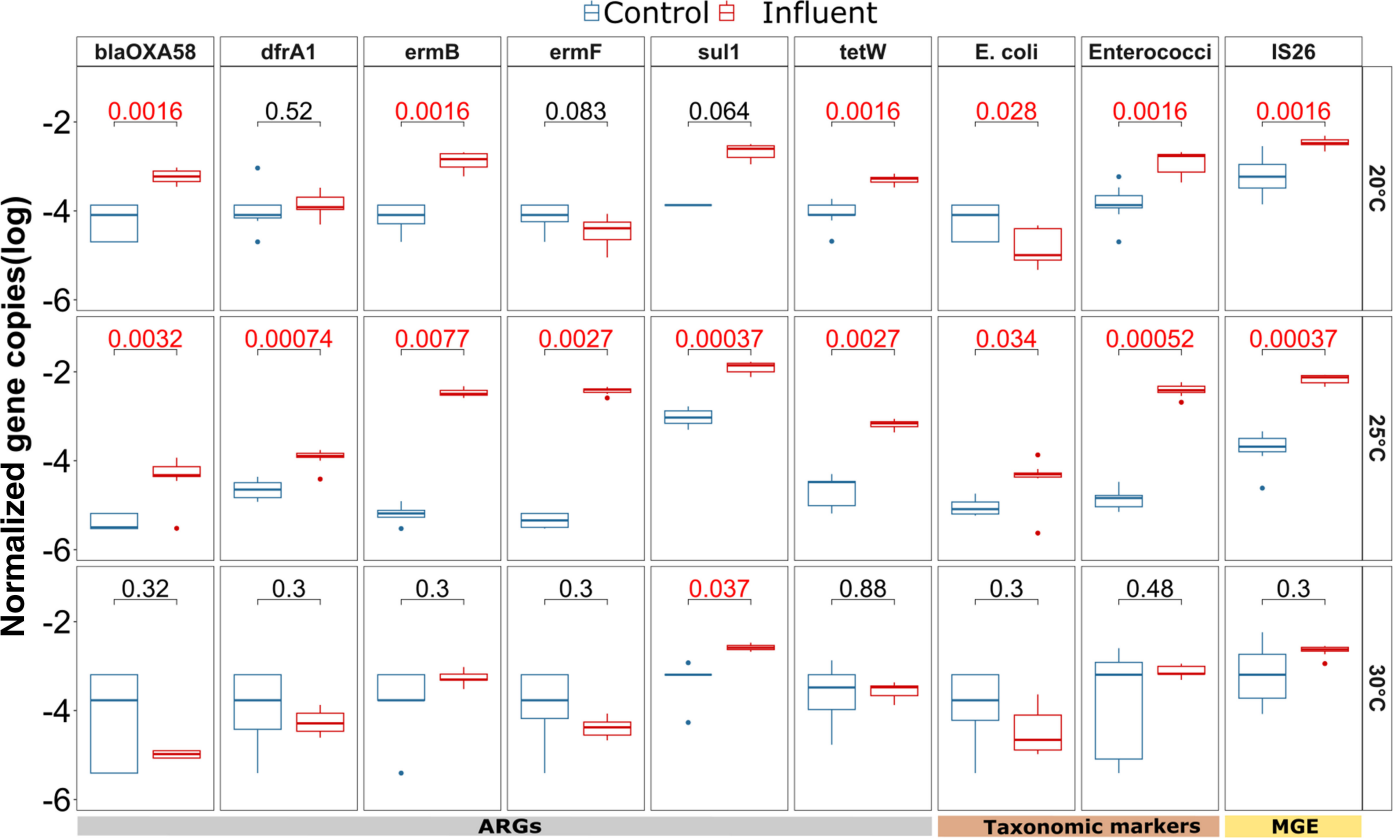
Indicator ARGs, taxonomic markers, and MGEs relative abundance in log scale on day 14 compared between the wastewater-invaded and the control biofilms across the three different temperatures (20°C, 25°C, and 30°C). The *P*-value (Wilcoxon test) of the comparison for each gene after correction for multiple testing is displayed at the top of each plot, with significant values (*P* < 0.05) displayed in red.

The temperature-dependent loss dynamics were also reflected in the outcome of the flume experiment. On day 14, at 30°C, the relative abundance of five out of the six invading ARGs and all three non-ARGs, the MGE *IS26,* and the two fecal indicator bacteria *E. coli* and *Enterococci* had returned to similar abundance level observed in the control flume. Only the indicator ARG *sul1* remained significantly elevated compared to the control group (*P* < 0.05, Wilcoxon test) (Fig. 5). On the contrary, at 20°C, three of the indicator ARGs (*blaOXA58, ermB,* and *tetW*), and at 25°C, all indicator ARGs remained significantly elevated (*P* < 0.01, Wilcoxon test). Similarly, the three non-ARG indicators remained at higher levels than those observed in the control at the two lower temperatures on day 14 (*P* < 0.05, Wilcoxon test).

Overall, this suggests that the loss of invading ARGs and organisms at 30°C is not only rapid but also that the river biofilm is resilient against these invaders and can return to its original level before invasion. On the contrary, at lower temperatures, the effects of the invasion event remain detectable for an elevated amount of time. Importantly, similar to the control flumes, no change in biomass was detected over the course of the experiment at either temperature again indicating that growth is a negligible factor for the observed temperature-dependent effects.

### Higher resilience of the microbial community composition with increasing temperature

**Fig 6:**
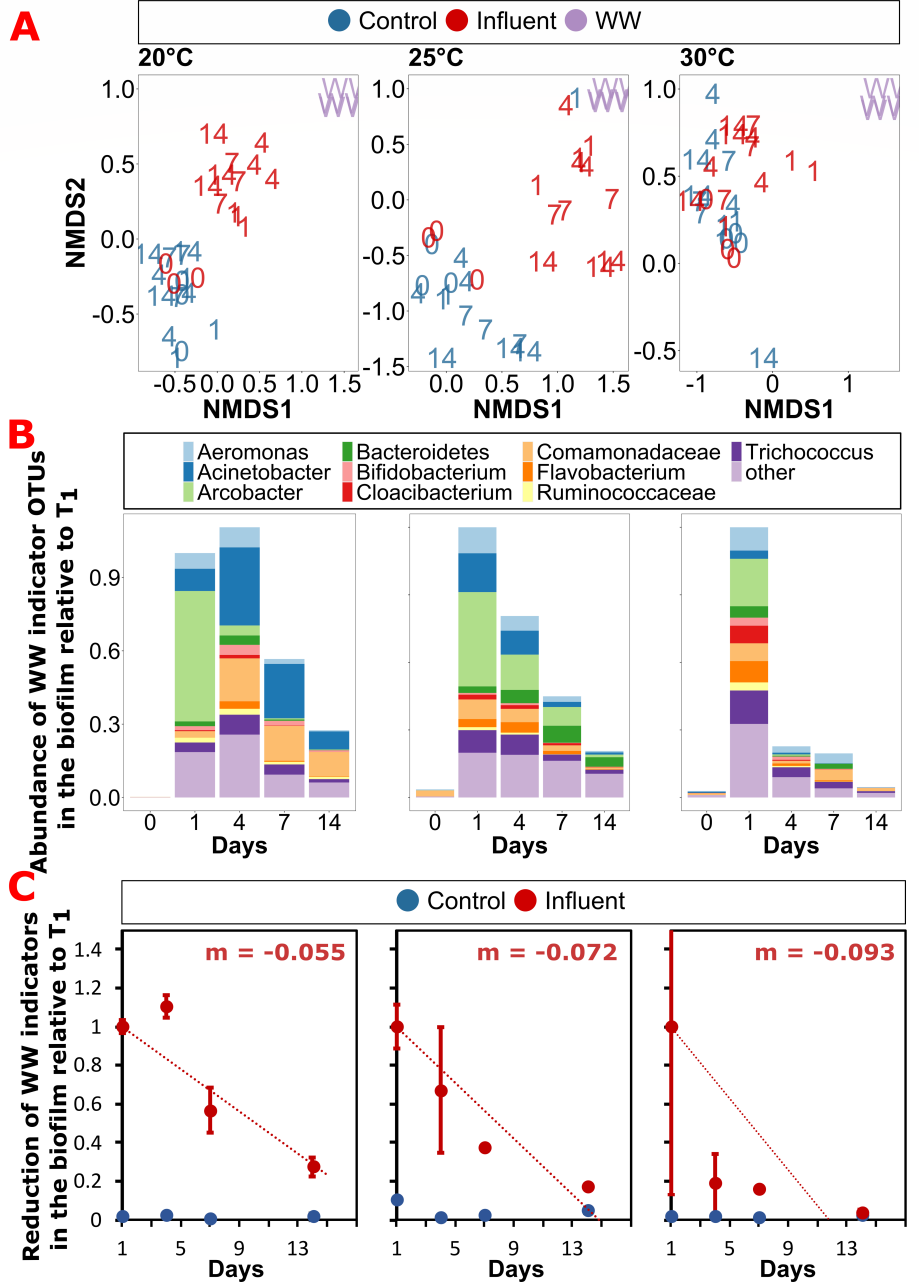
Microbiome diversity analysis. (**A**) Non-metric multidimensional scaling (NMDS) plot for beta-diversity of the river biofilm communities calculated using the Bray-Curtis dissimilarity at the OTU level. The NMDS plot for three temperature groups Tm20, Tm25, Tm30, and WW influent showing structural variations of the microbial community composition between the control and the treatment group. (**B**) Time-based dynamics of the wastewater invasion indicators in the invaded biofilms resolved at the OTU level. Abundance is normalized to the abundance of invaders detected at the respective temperatures. (**C**) Loss rates of wastewater invasion indicators over time at the respective temperatures.

To determine if the observed initial invasion into the biofilm with subsequent loss of invaders is not only reflected on the genetic but also on the community composition level, the microbial diversity dynamics over time of the biofilms were explored. For neither the controls nor the wastewater-invaded river biofilms, major changes in the phylum level distribution over time were observed, with *Pseudomonadota*, *Bacteroidota,* and *Bacillota* remaining the main observed phyla throughout (Fig. S4). However, on the beta-diversity level based on OTU abundances, the wastewater-invaded biofilms displayed major changes over time (Fig. 6A). Throughout, all biofilm samples cluster significantly apart from the initial invading wastewater community (*P* < 0.05, *R*^2^ = 0.294, ADONIS). Initially, the control and influent biofilms before treatment clustered together for all temperatures, and only minor changes in beta-diversity over time were observed for the control biofilms. However, on day 1 after the wastewater community was added to the flume, biofilms from the wastewater group clustered immediately between the initial biofilm as well as the initial wastewater samples but remained statistically different in diversity from either of the initial microbiomes (*P* < 0.05, *R*^2^ = 0.074, ADONIS). This trend was apparent across all three temperatures, suggesting indeed wastewater bacteria became part of the biofilm community and affected its diversity throughout (Fig. 6A). This was confirmed through analysis at the OTU level (Fig. 6B): 235 OTUs identified as originating from the wastewater community became part of the biofilm communities with their combined abundances on day 1 accounting for 43.4% ± 21.4 % of the total bacterial abundance with no apparent effect of temperature (*P* = 0.630, *R*² = 0.035, Pearson correlation). The top 10 invaders into the biofilms from wastewater belonged to the groups of ε- (*Arcobacter*), β- (*Comamonadaceae* spp.), and γ-Proteobacteria (*Acinetobacter* and *Aeromonas*), Bacteroidota (*Bacteriodetes* spp., *Flavobacterium*, and *Cloacibacterium*), and Bacillota (*Trichococcus* and *Ruminococcaceae* spp.). Over the experimental period of 14 days, the relative abundance of wastewater indicators normalized to the amount found on day 1 was significantly reduced at all temperatures by 72.5% (20°C), 82.8% (25°C), and 96.2% (30°C) (Fig. 6B; all *P* < 0.001, ANOVA). However, the reduction rate of these wastewater indicators per day was clearly correlated with temperature, where a significantly faster reduction back to the control levels within the experimental period was observed at 30°C, while wastewater indicator bacteria remained persistent in the two lower temperatures (Fig. 6C). The observed reduction effects were widely consistent across OTUs (Fig. 6B). These OTU-based results were also mirrored in the beta-diversity analysis, where at the two lower temperatures (20°C and 25°C), the wastewater-invaded biofilms, while significantly shifting in diversity, remained distinct and dissimilar to the initial biofilm, the control biofilms as well as the initial wastewater community. On the contrary, the wastewater-invaded biofilms at 30°C rapidly returned to cluster with the control biofilms, with the two treatment groups showing no significant distinction after 14 days when considering both, treatment and day in the ADONIS test (*P* = 0.12) (Fig. 6A). Hence, the previously detected resilience at the ARG level at the higher temperature is also realized at the microbiome level, with microbial diversity and composition returning rapidly back to the initial state after the invasion event.

## DISCUSSION

In this study, we demonstrate how increasing temperatures affect the dynamics of antimicrobial resistance in river biofilm communities. We observed contrary effects including an increase in the pre-existing natural ARGs present in the biofilm community and a decrease in the invasion success of foreign, wastewater-derived ARGs into the biofilms at elevated temperatures. Consequently, when estimating the effects of climate change on environmental resistomes, both these mechanisms need to be taken into account.

To determine the effects of warming on resistome dynamics, biofilms were grown on glass slides from a German river for several weeks, retrieved, and placed into laboratory flume recirculation systems at different elevated temperatures. As the sampling location is close to the spring of the river, and no wastewater or agricultural runoff is entering upstream, the biofilms can be considered as low anthropogenically impacted (57). Hence, any observed ARGs prior to wastewater exposure are likely part of the natural river resistome. Already after 1 week of acclimatization at the respective temperatures, biofilms in control flumes exposed to 30°C displayed a significantly higher relative abundance of overall as well as individual ARGs when compared to those exposed to 20°C and 25°C. This difference in ARG abundance remained throughout the experimental observation period of 14 days; however, no further increases with time were observed. In addition to the observed increases in resistance, communities of the different temperature treatments were significantly different. Similar trends of ARGs increasing with temperature were equally detected in long-term soil warming trials (58) and attributed to shifts in the microbial community.

From an ecological perspective, the observed dynamics can be explained based on the ARG hosts’ intrinsic fitness and/or the fitness of being resistant itself. First, if those bacteria hosting ARGs have a higher intrinsic fitness at higher temperatures, they would likely make up a higher relative proportion of the biofilm at elevated temperatures (59). In addition, the fitness cost of hosting ARGs is often temperature dependent, as resistance is not necessarily costly or selected against under thermal stress in the absence of any selection pressure through antibiotics (59–61). However, increases in the costs of resistance at higher temperatures have also been reported (62), indicating that effects might be highly dependent on hosts, ARGs, and the genetic context of the specific ARG. Our study suggests that the former, lower costs of resistance at higher temperatures is likely to be the predominant form in river biofilms, hence leading to the observed increase in ARGs.

While our study addresses increases in AMR with warming from an environmental, whole-community perspective, the observed effects are also mirrored in resistance profiles of specific pathogens on a clinical level (58, 63). Based on an ecological analysis of AMR prevalence in 4 million tested pathogens across 28 European countries between 2000 and 2016, countries with warmer ambient minimum temperatures experienced more rapid resistance increases ranging from 0.33% to 1.2% per year across all antibiotics (63). Furthermore, in a study across 28 Chinese provinces between 2005 and 2019, a 1°C increase in average ambient temperature was associated with a 6%–14% increase in carbapenem resistance in *Klebsiella pneumoniae* and *Pseudomonas aeruginosa* (58). These effects held true, even after accounting for known drivers of resistance such as antibiotic consumption and population density.

Aside from temperature effects on the natural resistome of river biofilm communities, the invasion dynamics of ARGs from a wastewater community into these river biofilms at three different temperatures (20°C, 25°C, and 30°C) were studied. Successful initial invasion of resistant bacteria and ARGs into the biofilm was observed shortly after their introduction to the flumes on day 1. The previously discussed increases in ARGs due to temperature were relatively minor compared to those observed due to invasion from wastewater, which were orders of magnitude higher, suggesting that in heavily wastewater-impacted rivers, invasion is the main process of concern with regards to AMR. Specifically, six ARGs, one MGE, and two taxonomic markers were detected at higher relative abundance than in the control biofilms, with relative abundances increasing by several orders of magnitude across all temperatures. Among the nine invasion indicator markers, the identified ARGs are well known to be associated with anthropogenic activities and WWTPs (13, 64, 65) and were also observed in our wastewater sample at far elevated abundance compared to the original biofilms. The taxonomic markers, *E. coli* and *Enterococci,* are well-known fecal pollution indicators (13, 26), and multi-drug resistance has been regularly reported for both (66). Therefore, *E. coli* and *Enterococci* can be considered as model invading resistant bacteria for this study.

With regards to temperature, on day 1, no clear distinct pattern regarding the initial invasion success of the nine invasion indicators was observed between the temperature groups. This suggests that the initial introduction of the invaders to the biofilm mainly depends on the propagule pressure, hence the number of invading bacteria or genes (67, 68). This coincides with the result from a previous study using the same setup where the biofilms were invaded by *E. coli* in the presence and absence of copper-induced stress, and the initial invasion success of *E. coli* entering the biofilm was purely stochastic and independent of stress (33). Furthermore, the successful initial invasion was equally observed in the community composition of the biofilms based on beta-diversity and OTU-level analysis of wastewater invasion indicator OTUs. Day 1 biofilm communities consistently grouped between the original biofilm and the wastewater samples with around 40% of the relative abundance accounting for wastewater invasion indicator OTUs, confirming that invasion into the biofilm was detectable on the microbiome level.

Over time, after the initial invasion event, a decline in the relative abundance of the invasion indicators at both the gene as well as the OTU level was observed for all temperature groups. However, this decline was far more rapid and pronounced for all markers at the highest temperature (30°C). This resulted in indicator levels at 30°C returning to similar levels than those of the non-invaded control biofilms after 14 days. On the contrary, the majority of indicators remained significantly elevated compared to the control biofilm at 20°C and 25°C. Considering that at the gene level, the natural levels of ARGs in the 30°C flumes were significantly elevated, as discussed earlier, it is important to note that the levels observed in the invaded 20°C and 25°C biofilms remained above the levels obtained in the 30°C control flume. Similar dynamics were apparent with regards to community composition, where no difference in diversity or wastewater invasion indicator OTU levels between the control and the invaded biofilms was observed at 30°C after 14 days, while at 20°C and 25°C, the communities of the biofilms remained significantly different and invader levels remained elevated. Consequently, the river biofilms display an increased level of resilience toward invasion at higher temperatures.

When considering these results of invasion events within the framework of community coalescence, it can be expected that invading species replace indigenous species, if they are better adapted to the prevailing environmental conditions (69–71). Considering that many of the resistant wastewater bacteria are of fecal origin, their temperature optima are likely to be higher than those of the indigenous river biofilm community. For example, for the two model invader species monitored through qPCR, the temperature optimum of *E. coli* is 37°C–42°C (72) and for *Enterococci* is 42°C–45°C (73), which would suggest that their invasion success should be higher at elevated temperatures. However, higher temperatures also increase the metabolic rates of the indigenous community members, hence leading to a faster depletion of resources, resulting in a higher level of competition through limited resource access toward the invading species. This increased competition, together with the fact that many fecal bacteria struggle to display significant growth rates under environmental nutrient conditions independent of temperature (74, 75), could lead to the observed faster elimination of invading bacteria and their ARGs. In addition, priority effects, which regularly determine the outcome of coalescence events (76), could here be at play as the indigenous biofilm is already established before invasion. Such priority effects could play an increased role when the biofilm bacteria show an increased level of activity at higher temperatures. The here-applied short-term changes in temperature could, in theory, also lead to an altered and less stable microbial community structure, making priority effects obsolete (77). Still, in our experiments, biofilms were left to acclimatize to the temperature for 1 week before inoculation and remained stable in composition thereafter. This suggests that the community adapted to the respective temperatures and can be considered as established, meaning priority effects including an inherent advantage against invading bacteria are at play. Together, these factors contribute to the observed dynamics, where a higher resilience of the river biofilm community toward invasion by resistant bacteria and their ARGs is observed at 30°C. Similar effects on the temperature dependence of coalescence in aquatic bacterial communities were observed on the microbiome level when mixing marine communities across different temperatures, where resistance of aquatic bacterial communities to invasion was shown to be strengthened by warming (78). This allows us to presume that the here-observed effects regarding AMR are likely to be transferable to other scenarios where invasion takes place.

Notably, the temperature effects described here did not follow a linear trend with increasing temperature. While consistent significant differences between the 30°C and the two lower temperature treatments were observed, the biofilms at 20°C and 25°C behaved rather similarly with regard to the levels of natural resistance in control biofilms as well as invasion dynamics of foreign ARGs from wastewater. Temperature dependencies of microbial processes or ecosystem services regularly do not follow linear trends, as the influence of temperature on them is mediated by the temperature sensitivity of the individuals making up the microbial community and their biotic interactions (79, 80). Rather, temperature thresholds exist at which the temperature dependence switches between low, intermediate, and high temperatures, as has, for example, been demonstrated for global ecosystem respiration rates (81). Our results suggest that temperature effects regarding the spread of resistance in river microbial communities might equally follow a threshold rather than a linear temperature model.

Overall, we here demonstrate the contrary effects of increasing temperature on the dynamics of AMR in river biofilm communities. While an increase in natural resistance in the biofilm at elevated temperatures was apparent, it coincided with a decrease in invasion success of foreign ARGs originating from wastewater. This implies that estimating the effects of climate change on river microbial communities might highly depend on which of the two processes dominates in the specific river ecosystem. Within this context, it is important to take into account that most rivers are heavily anthropized and receive daily wastewater influx. Hence, the temperature-improved barrier effect of natural systems against invading ARGs and species, here observed for a single wastewater pulse, might in, general, be low and decreasing with time due to the constantly happening invasion events. Future research into how this barrier effect evolves over time and with constant wastewater inflow is hence necessary to further and more accurately predict climate change effects.

## Data Availability

The main data sets supporting the conclusions of this article are included within the article. Original sequencing data are available in the NCBI sequencing read archive under project accession number PRJNA1014398. Any additional data are available through the corresponding author upon reasonable request.

## SUPPLEMENTAL MATERIAL

Supplemental Material msphere.00573-23-s0001.docx Figures S1-S4 and Table S1.

ASM does not own the copyrights to Supplemental Material that may be linked to, or accessed through, an article. The authors have granted ASM a non-exclusive, world-wide license to publish the Supplemental Material files. Please contact the corresponding author directly for reuse.
